# Can Immune Therapy Cure Acute Myeloid Leukemia?

**DOI:** 10.1007/s11864-023-01066-3

**Published:** 2023-03-22

**Authors:** Robert Peter Gale

**Affiliations:** Centre for Haematology, Department of Immunology and Inflammation, Imperial College of Science, Technology and Medicine, London, SW7 2BX UK

**Keywords:** Acute myeloid leukemia, Mutations, Neo-antigens, Immune therapy, Gemtuzumab

## Abstract

Although safe and effective immune therapies have been developed in several cancers, this has not been so in acute myeloid leukaemia (AML). Studies of antibodies to CD33, CD123 and CLL-1 report with unconvincing efficacy and substantial adverse events. Lacking AML-specific target antigens, these approaches using non-specific antigen targets often cause unacceptable bone marrow toxicity and off-target adverse events. Studies of AML incidence in persons with immune deficiency indicate little if any immune surveillance against AML. In contrast, data studies of recipients of haematopoietic cell transplants support an effective allogeneic anti-AML effect associated with graft-*versus*-host disease (G*v*HD) and possibly a specific graft-*vers*us-leukaemia (G*v*L) effect. A special problem in the immune therapy of AML is few neo-antigens compared with solid cancers because of a relatively low mutation frequency. Studies of CAR-T-, CAR-NK-adaptor CAR-T- and allogeneic NK-cells are progressing as are approaches using synthetic biology. Presently, there are no convincing data of efficacy of immune therapy in AML.

## Introduction

Forty years ago, we reviewed the role of immune therapy in AML [1]. We interrogated data from 24 clinical trials in almost 1500 subjects. Interventions included Bacillus Calmette-Guerin (BCG) and methanol extract residue (MER) of BCG, *Corynebacterium parvum*. Some subjects received allogeneic or autologous leukaemia cells. We concluded immune therapy was ineffective in AML.

In the past 10 years, immune therapy has been developed in B-cell and solid cancers [2•, 3, 4•]. In haematology, safety and efficacy of immune therapy are predominately in B-cell lymphoid cancers where monoclonal antibodies, antibody–drug and -radionuclide conjugates, bi-specific antibodies and chimeric antigen receptor T-cells (CAR-T-cells) have proved useful. These immune therapies target B-cell lineage rather than cancer-specific antigens. Checkpoint inhibitors active in solid cancers are relatively ineffective in haematological cancers. These considerations raise the question of why there is so little progress in immune therapy of acute myeloid leukaemia (AML).

### The difference between lymphoid cancers and AML

Lymphoid cancers and AML are different in several aspects. For example, only about 10 million lymphoid cells are produced daily compared with about 600 billion myeloid cells. Also, granulocytes and platelets survive only a few hours or days compared with lymphoid cells which live years. The consequence is a disruption of myelopoiesis is much more dangerous than a disruption of lymphopoiesis. Second, this is the different targetability of lymphoid compared with myeloid antigens. The target of immune therapy of lymphoid cancers is B-lineage antigens which are not cancer specific. Killing substantial numbers of normal B-cells is compatible with life, whereas this is not so for killing substantial numbers of myeloid cells. Consequently, immune therapy of AML is limited by the absence of AML-specific target antigens and potential damage to normal bone marrow function.

### Is there immune surveillance against AML?

**Fig. 1:**
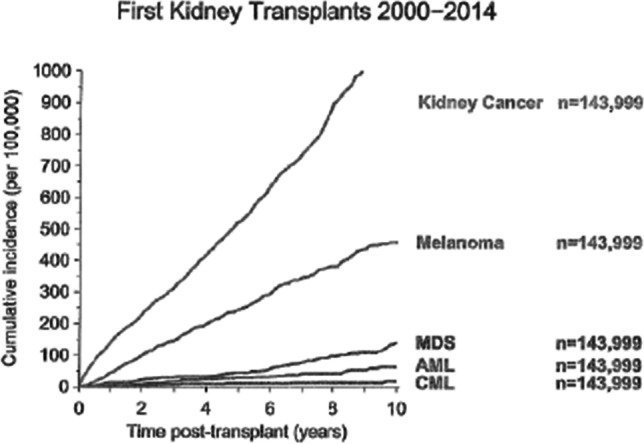
Infrequent AML in kidney transplant recipients [5]

Risks of lymphomas, melanoma and kidney cancer are substantially increased in lymphoma risk in persons with immune deficiency or iatrogenic suppression. In contrast, there is only a small increased risk of AML in these settings suggesting immune surveillance does not operate in AML (Fig. 1) [5].

### Is there an immune response to AML?

**Fig. 2:**
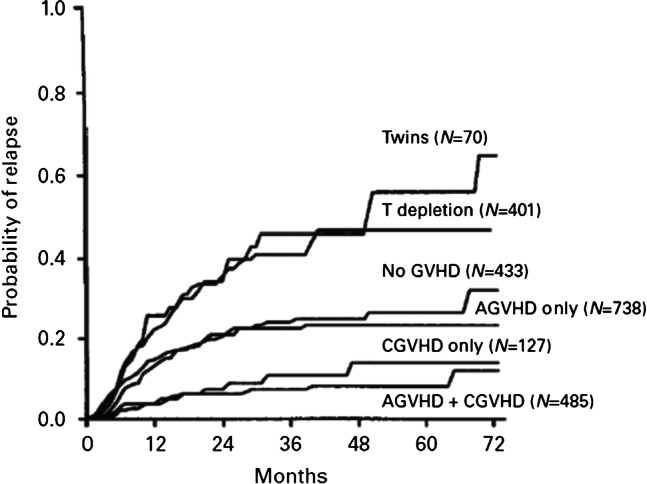
Probability of leukaemia relapse after allotransplants for AML adjusted for subject- and leukaemia-related co-variates [6]

In another study, we evaluated the probability leukaemia relapse in persons with AML receiving haematopoietic cell transplants from different donor types, different graft contents and different graft-*versus-*host disease (G*v*HD) outcomes (Fig. 2) [6]. After adjusting for subject- and leukaemia-related co-variates, recipients of transplants from genetically identical twins had the highest relapse probability, whereas those with acute and chronic G*v*HD had the lowest relapse probability. In the context of allogeneic haematopoietic cell transplants, there are several potential anti-AML target antigens including HLA antigens, minor histocompatibility antigens, leukemia-associated antigens and (4) leukemia-specific antigens. Whether this allogeneic graft-*versus*-leukemia effect (GvL) is distinct from GvHD is controversial [7].

### What is the role of AML-specific antigens in graft-*versus*-leukaemia (G*v*L)?

**Fig. 3:**
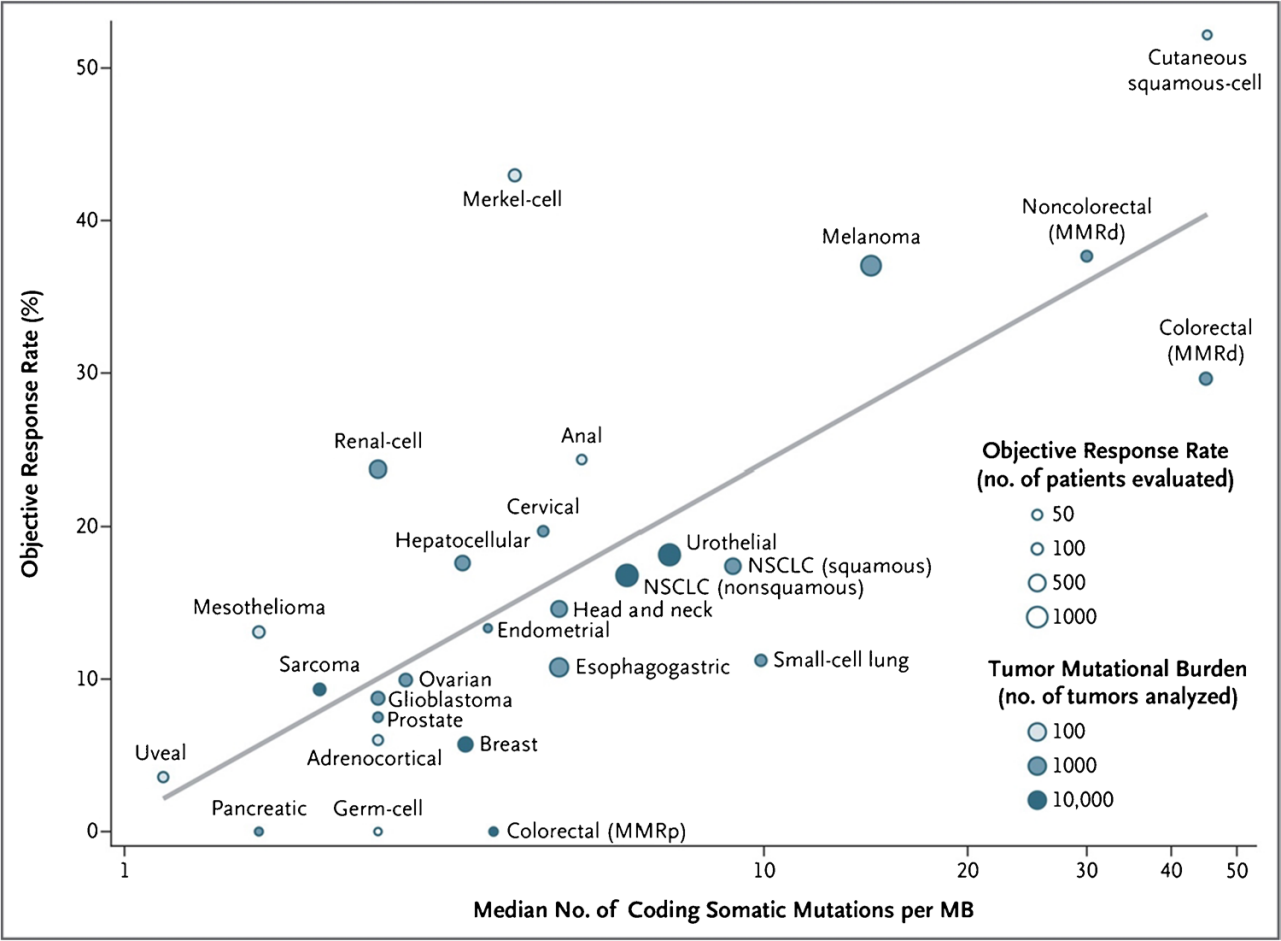
Correlation between coding somatic mutation frequency and objective response rates in diverse cancers [8]

Several variables correlate with success of immune therapy including antigenicity, immunogenicity, accessibility, sensitivity to killing and collateral damage to normal cells. In AML, we are faced with the lack of a AML-specific target antigen(s). In solid cancers, efficacy checkpoint inhibitor antibodies correlate with mutation frequency (Fig. 3) [8]. However, compared with the average solid cancer, AML cells have 40–100-fold fewer mutations per megabase of DNA. Clinical studies of immune checkpoint inhibitors like anti-PD-1 antibodies report little or no benefit [9, 10].

### Clinical trials

Several immune therapies have been studied in AML including monoclonal antibodies, drug-, -toxin and radionuclide antibody conjugates, bi-specific antibodies and others (Table 1) [11, 12].

**Table 1:** Antibody-based therapies of acute myeloid leukemia

Antibodies	CD33	Lintuzumab
	CD38	Daratumumab
ADC	CD33	Gemtuzumab, SGN331, IMGN779
	CD123	SL-401, SGN-CD123A
RIT	CD33	^225^Ac-Lintuzumab
	CD45	^131^I-BC8
Bi-specific	CD33	AMG330
	CD123	Flotetuzumab, JNJ-637099178
Checkpoint	PD-1/-1L	Nivolumab, pembrolizumab, avelumab
	CTLA-4	Ipilimumab

### Cell-based immune therapy

Studies of cell therapies in AML include NK- and CAR-T- and CAR-NK-cells and cytokine-induced NK-cells (CIK) [13•, 14]. There are as yet no convincing data of safety or efficacy [15, 16]. Synthetic biology techniques may allow use of anti-CD33 antibodies [17].

## Conclusions

Compared with immune therapy of B-cell lymphomas and several solid cancers, there has been little progress in immune therapy of AML outside the context of an allogeneic haematopoietic cell transplant. The predominant challenges are the lack of an AML-specific target antigen(s) and presently unavoidable collateral damage to normal bone marrow function. Whether these challenges can be overcome is unknown.
